# Enhancing Cognitive Care Planning: Healthcare Providers’ Insights on Paper-Based vs. Digital Tools

**DOI:** 10.1093/geroni/igaf122.1793

**Published:** 2025-12-31

**Authors:** Shaoqing Ge, Xaviera Xiao, Katherine Britt, Bin Huang

**Affiliations:** University of Texas at Austin School of Nursing, Austin, Texas, United States; Brain Check, Austin, Texas, United States; University of Iowa, Iowa City, Iowa, United States; BrainCheck Inc, Austin, Texas, United States

## Abstract

Cognitive care planning (CCP) is an effective intervention to manage and improve health among patients with cognitive impairments like Alzheimer’s Disease and Related Dementias (ADRD). Despite its benefits, CCP is underutilized in clinical settings. The Alzheimer’s Association (AA) has developed paper-based guidelines to promote CCP. Online tools are also emerging to support healthcare providers and patients in implementing CCP, automatically generating assessment results and care plans. This study examines the perceptions and impact of a comprehensive online platform compared to AA’s paper/pdf-based guideline through 17 interviews with healthcare providers, focusing on providers’ perceptions of using these tools in clinical settings. Our findings demonstrate several perceived advantages of the all-inclusive digital cognitive assessment and care planning tool. Such advantages include the necessity and value of a computerized assessment that can be easily adapted to existing electronic medical record systems. Additionally, the time-saving potential of an automated assessment and automatically generated care plan was also mentioned. Healthcare providers also mentioned challenges that may arise from an all-inclusive platform wherein assessments and surveys are carried out uniformly amongst patient populations, causing concerns surrounding the lack of nuance and “humanness”. Finally, concerns about technology acceptance amongst older adult patients and older adult caregivers and reimbursement-related issues were also highlighted. In conclusion, the digital cognitive assessment and care planning tool is acknowledged by providers as beneficial for promoting CCP. Our study provides insights into the impact of digital platforms in supporting older adults with cognitive decline and AD/ADRD and their caregivers in clinical care settings.

